# Determinants of optimum exclusive breastfeeding duration in rural India: a mixed method approach using cohort and content analysis design

**DOI:** 10.1186/s13006-021-00359-3

**Published:** 2021-01-21

**Authors:** Falguni Debnath, Nilanjan Mondal, Alok Kumar Deb, Debjit Chakraborty, Subhrangshu Chakraborty, Shanta Dutta

**Affiliations:** 1 ICMR-National Institute of Cholera and Enteric Diseases, Kolkata, India; 2 Department of Health & Family Welfare, Government of West Bengal, Kolkata, West Bengal India

**Keywords:** Exclusive breastfeeding, Insufficient nutrition, Interruption of EBF, Facilitators, Family support, Cultural belief, Feeding preferences

## Abstract

**Background:**

Despite established benefits, exclusive breastfeeding (EBF) rate remains poor in India. This study measured the rate of early initiation of breastfeeding and EBF up to 42 days postpartum period and the reasons associated with early interruption of it.

**Methods:**

In this study we followed a cohort 319 mother-newborn dyads, on a scheduled day of each week for six postpartum weeks (42 postpartum days), during May 2017 – March 2019. We used standard maternal 24 h recall method to collect data on newborn feeding practices. Additionally, using content the analysis method, we analysed the data captured through open ended question on current breastfeeding practice and reasons to identify the sociocultural facilitators/barriers of exclusive breastfeeding .

**Results:**

Of the retained 306 newborns, early initiation of breastfeeding rate was 60% (184/306), whereas, EBF rate was 47% (143/306). Mothers’ educational level did not emerge as a risk for unsuccessful breastfeeding practices, whereas, father being not the major earner of the family (Relative risk [RR] 2.4; 95% Confidence interval [CI] 1.7,3.3), mothers who did not believe that effect of breastfeeding is longstanding (RR 1.8; 95% CI 1.3, 2.1) emerged as a risk for unsuccessful EBF practices. Lack of self-conviction about EBF among mothers; significant family members’ influence; cultural beliefs; emerged as major socio-environmental barriers of early interruption of exclusive breastfeeding. Repeated counselling by the healthcare provider particularly focusing on exclusive breastfeeding, supportive family environment in terms of the elders being aware of the positive health outcomes of it, and prior positive experience emerged as the socio-environmental facilitators for successful EBF until 42 postpartum days.

**Conclusions:**

We conclude that the socio-environmental causes need to be addressed through the present healthcare delivery system for ensuring better infant feeding outcome.

## Background

India, where more than half of the population reside in rural areas, has come a long way in terms of providing minimum antenatal care (at least four antenatal care visits) visits from 21% during the year 1992–93 to 45% in 2016, but the breastfeeding rates have not been impressive so far. According to National Family Health Survey 4, 41.6% children under 3 years of age were breastfed within an hour of birth [1], whereas 54.9% infants under 6 months of age were exclusively breastfed [1] and 38.4% under-five children were stunted. The median duration of exclusive breastfeeding is 2.9 months in India, whereas, median duration of predominant breastfeeding is 5.8 months [2]. The Global Burden of Disease Study 1990–2017 states that based on the modest increasing trends between 1990 and 2017, the projected prevalence of exclusive breastfeeding for India is 59.3, 10.7% less than the WHO and UNICEF 2030 target of at least 70% [3].

West Bengal has documented that 52.3% of its under 6 months old infants were exclusively breastfed [2], the proportion of early initiation of breastfeeding was lower (48.2%) [2]. In this state, the median duration of exclusive breastfeeding is 2.6 months, whereas the neighbouring states Odisha and Jharkhand reported a median duration of 4 months for exclusive breastfeeding [2]. However, not many studies have explored the socio-cultural, familial, environmental issues at local context for having such low rate of exclusive breastfeeding [3].

Hence, we conducted a cohort study with the following objectives.

### Primary objectives

1. To estimate the proportion of newborns who initiated early breastfeeding after birth.
2. To estimate the proportion of infants exclusively breastfed up to 42 days postpartum period.

### Secondary objective

To understand the deterministic factors and barriers associated with early interruption of exclusive breastfeeding.

## Methods

### Study settings

We conducted this study at Dhaniakhali Community Development Block of Hooghli district. The total population is 320,534 and representative of existing rural healthcare system. We recruited the mothers who delivered at that hospital and were going to reside in that block for next 42 days. The mothers willing to participate were included after obtaining written informed consent. We did not recruit any clinically unstable mother or newborn in our study. The recruitment was done 24 h post-delivery.

### Sample size

We needed to recruit 319 mother–newborn dyads, assuming that 38.3% (National Family Health Survey 4) [2] of them are initiated early initiation of breastfeeding, odds ratio [OR] of two, 80% of power, 95% confidence interval and accounting for 10% of non-response.

### Data collection

We collected data using a semi structured questionnaire on sociodemographic details; practices regarding breastfeeding and their reasons; mothers’ knowledge and attitude regarding breastfeeding; delivery related issues, newborn information. Open-ended questions were specifically asked for current feeding practices and [4] by asking her what had been fed to the newborn in last 24 h as this is the standard practiced method [4].

### Follow up schedule

Post recruitment, weekly visits (starting from 1st week of life) were made for all the recruited mother-child dyads up to 42 days’ postpartum period by the community health workers. We limited the follow-up till 42 days postpartum period as this activity was within the scope of current healthcare system activity. Establishing a parallel system for following the mother-child dyads for 6 months would not have been a sustainable process, hence, we focused on using the available resource within the scope of current healthcare system for recommending feasible and sustainable changes.

### Definitions

Early initiation of exclusive breastfeeding: We defined early initiation of breastfeeding as started breastfeeding the newborn within 1 h of birth [4, 5].

EBF: We defined exclusive breastfeeding as breastfeeding without the introduction of other food or liquids (not even water) over the prior 24-h period, with the exception of drops or syrups consisting of vitamins, mineral supplements or medicine [4, 5].

Predominant breastfeeding: We defined it as if the infant receives plain water or water-based liquids such as juice along with breastfeeding [4, 5].

Partial breastfeeding: The inclusion of other milks, infant formula, semi­solids along with breastfeeding was defined as partial breastfeeding [4, 5].

### Data analysis plan

#### Quantitative data analysis

Quantitative data on sociodemographic details, rates of early initiation of breastfeeding and exclusive breastfeeding were summarized using descriptive statistics. We computed the risks along with 95% of confidence interval (CI) for failure of exclusive breastfeeding for first 42 days of the infants’ life.

#### Qualitative data analysis

The qualitative data collected through asking open ended questions were analysed obtaining manual content analysis method and presented the information through abstraction matrix [6]. Answers to open ended questions were included till the answers got saturated.

### Ethical statement

This study was approved by the institutional ethics review committee of National Institute of Cholera and Enteric Diseases, Kolkata, India and we recruited the participants after obtaining written informed consent.

## Results

### Baseline characteristics of the study participants

Out of 319 recruited mother-newborn dyads, we retained 306 dyads.

The mean age of the recruited mothers was 22.6 years (Standard deviation, 3.7 years) (Table 1). Except for three newborns, all were delivered vaginally and only 40 (13%) newborns weighed less than 2.5 Kg. Less than 50 % of the recruited mothers gave birth for the first time. More than one third of the recruited mothers belonged to Schedule Tribe community (SC). Only 31% (93) of the mothers were educated beyond primary level and 11% (35) of them were illiterate. Half of the mothers live in a joint family and only in more than one third families, the father of the newborn was the major earner of the household (Table 1).

**Table 1:** Baseline characteristics of study participants: exploratory study of reasons for failure of EBF at 42 days’ postpartum period, Hooghli, West Bengal, India, 2018 (*N* = 306)

Characteristics		***n***	%
Age	≤22	159	52
	> 23	147	48
Religion	Hindu	270	88
Caste	ST	110	**36**
	SC	138	**45**
Educational qualification of the mother	Up to primary	178	**58**
	Illiterate	35	**11**
Educational qualification of the father	Up to primary	198	**66**
	Illiterate	54	**18**
Socioeconomic status	Lower	261	**85**
Family type	Joint family	164	**53**
Families where father of the child is the major earner		105	**34**
BMI	< 18.5Kg/m^2^	35	**11**
	23–24.9 & ≥25 Kg/m^2^	111	**36**

### Rates of early initiation of breastfeeding and EBF up to 42 days of postpartum period

All the newborns received colostrum after delivery. Out of all the recruited 306 newborns, 60% (184/306) newborns were initiated breastfeeding within 1 h of birth. The median time taken to start breastfeeding was 40 min (IQR, 34–81 min). Whereas, 143 (of 306, 47%) infants were exclusively breast fed up to 42 days postpartum period.

**Fig. 1:**
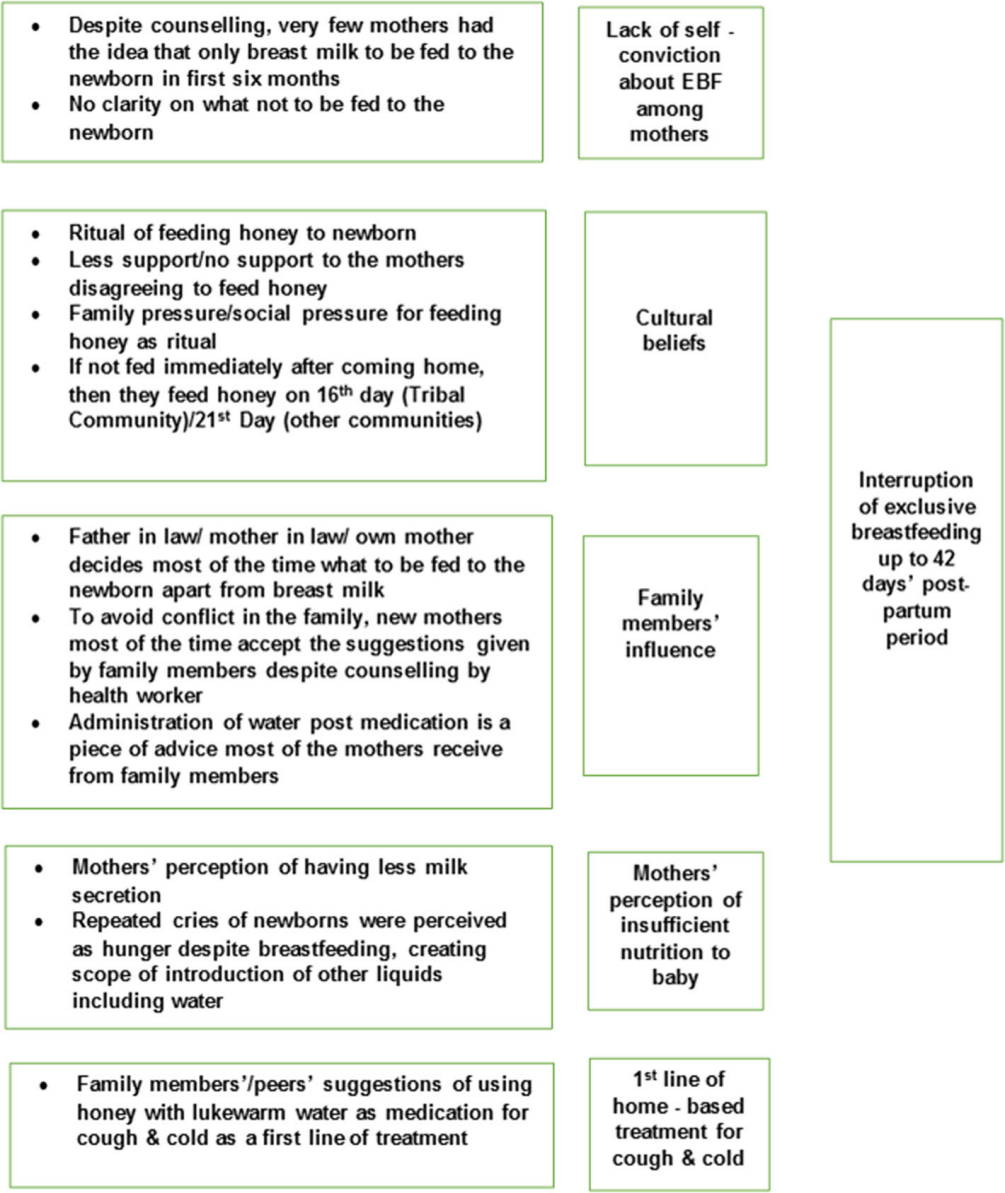
Socio-cultural reasons of interruption of exclusive breastfeeding in first few weeks of life in Hooghli, West Bengal, India (*N* = 169). Legend EBF: Exclusive breastfeeding

Out of the 163 newborns, who were not exclusively breastfed, 20% (33) could be classified as predominantly breastfed, whereas, rest 81% (133) newborns could be classified as partially breastfed (Fig. 1).

### Risks of unsuccessful exclusive breastfeeding practices for first 42 days of infants’ life

Mothers’ educational level did not emerge as a risk for unsuccessful breastfeeding practices, whereas, father being not the major earner of the family (Relative Risk [RR], 2.4; 95% Confidence Interval [CI], 1.7, 3.3), mothers who did not believe that effect of breastfeeding is longstanding (RR 1.8; 95% CI 1.3, 2.1) emerged as a risk for unsuccessful EBF practices (Table 2).

**Table 2:** Risks for unsuccessful exclusive breastfeeding practices for first 42 days infants’ life, West Bengal, India, 2017–2019 (*N* = 306)

Characteristics		Outcome				Relative Risk	95% Confidence Interval
		Unsuccessful EBF (163)		Successful EBF (143)			
		n	%	n	%		
Mother’s education	Illiterate (35)	18	51.4	17	48.6	1.2	0.6, 2.1
	Primary to high school (247)	135	54.7	112	45.3	1.3	0.8, 2.1
	Higher secondary till post graduate (24)	10	47.5	14	52.5	1	
Father the major earner of family	No (201)	134	66.7	67	33.3	**2.4**	1.7, 3.3
	Yes (105)	29	27.6	76	72.4		
Mothers who believe that effect of breastfeeding is longstanding	No	68	77.3	20	22.7	**1.8**	1.5, 2.1
	Yes	95	43.6	123	54.4		
Mothers who believe that locally available food can be started from 2 to 3 months onwards	Yes (58)	40	69	18	31	**1.4**	1.1, 1.7
	No (248)	123	49.6	125	50.4		
Mothers who heard breastfeeding reduces chance of breast related disease	No	135	65.5	71	34.5	**2.3**	1.6, 3.2
	Yes	28	28	72	72		

### Socio-environmental barriers of exclusive breastfeeding up to 42 days of postpartum period

#### Lack of self - conviction about breastfeeding among the mothers (Fig. 1)

Of all the recruited mothers, only four mothers could not express their views about “exclusive breastfeeding”. Hundred and fifty-nine mothers told that they need to feed breast milk to the baby for at-least first 6 months but only very few mothers (*n* = 3) mothers knew that only breastmilk should be fed to the infant for the first 6 months. Most of the mothers reported that though they have been told to breastfeed their newborn, but they did not have clarity on what else not to be fed.

#### Cultural belief (Fig. 1)

In most instances, on probing, mothers accepted feeding of other liquids predominantly “honey” to their newborns especially during the first 2 weeks. “Feeding honey” was treated as a welcome gesture to the newborns, practiced either on 16th day (ST community) or 21st day post birth (SC/general Bengali community). Many of the mothers justified this as an important cultural ritual, whereas, many highlighted poor controls over ritualistic practices run in the family, showcasing the strength of role of the cultural believes in rural Indian settings. For example, a participant of 25 years expressed the reason for feeding honey to the child as “*This is my second child and is a son; my husband is so happy, he only fed honey after coming home. He was so happy that he gave it first and then my in-laws, neighbours also fed the same*”. Another mother from lower socio-economic section highlighted the aspect of poor power over choice of food to be fed to the child by saying “*This is a ritual, we have to give, if I disagree then also family members will give. Better not to disagree. We do not do all these in hospital, Sister didi (Nurse) told not to give. Family members wanted to feed her honey, so they fed*” (19 years old mother who belong to SC community).

#### Family member’s influence on choice of feeding mode (Fig. 1)

Introduction of cow milk apart from honey and water during as early as second week of life was an important observation. Apart from cultural believes, family member’s influence on choice of feeding mode emerged as a one of the major reasons for introduction of other liquids to the newborns. An 18 years old mother from ST community living in a joint family structure, whose husband was not the major earner of the family said “*Family members (Father in law said but mother in law also agreed) suggested to do so, now feeding little cow milk with water and breast milk also. I should not deny. They know more*”. Post medication administration of water was another piece of advice, received from family members.

#### Mother’s perception of insufficient nutrition (Fig. 1)

Mother’s perception of having insufficient breast milk was another reason of introduction of other liquids to the newborns. Strength of this perception can even be appraised as the experienced mothers also could not get rid of it. For example, a 24-year-old female participant who delivered for the second time said, “*I feel I am having less milk, I was feeding cow milk, but my neighbour told to feed formula, they bought a packet also. So, now stopped giving cow milk, giving formula now*”. But unfortunately, none of them sought medical advices. Even in many instances repeated cries of the babies were misperceived as thirst and water was fed to them.

### Facilitators of exclusive breastfeeding practice up to 42 days’ postpartum period

The mothers, who managed to feed only breast-milk to their newborns highlighted the importance of repeated counselling by the healthcare provider particularly focusing on exclusive breastfeeding, a supportive family environment where the elders were aware about the positive health outcomes and prior positive experience. A 26 years old participant who became mother for second time said” *During the time of my first child, doctor told to give breast milk only for 6 months. I know this, I will do the same for this child as well*”. Even a mother in law of a 20 years old added to her daughter-in-laws’ statement by saying,” *We do not give anything to babies other than breastmilk for 6-7 months from birth*”. Repeated counselling from healthcare providers particularly focusing on EBF showed a positive effect in our study which can be understood by the verbatim of a 23 years old mother who belongs to schedule cast community, “*I will feed only breast milk up to six months, Didi (Auxiliary nurse midwife) told me repeatedly before delivery. I know, breastmilk is only good for my baby. Even if I start going to work, my family members will take my baby to the field for a feed*.” Self-conviction about the positive health outcomes of exclusive breastfeeding emerged as another reason for successful EBF till 42 days postpartum period. “*Breastmilk is the only good food for baby up to six-months, even won’t give water* ‘, said by a 28-year-old mother who delivered for the first time.

## Discussion

In this paper, we described how the globally desired goals for EBF hits reality in the first few weeks of life. Despite collective efforts, the progress in this issue is much slower in India. Our study explored the reasons and found that though majority of the study population belong from two different communities but the reasons for failure of exclusive breastfeeding during first few weeks of life were prevalent across communities.

Cultural influences and beliefs about breastfeeding and infant nutrition emerged as a strong reason for early interruption of exclusive breastfeeding even as early as in the first week of life. In this study, mothers justified the introduction of other liquids in the name of “important ritual”, whereas, a section of mothers expressed the issue of family conflict in case of disagreement to the family customs or traditions, highlighting the importance of preparing important family members along with the pregnant mothers for ensuring better infant feeding outcomes. The women from the poorer section of the community usually have very less contribution in family decisions. In our study, we found in only one third families, the father of the newborn was the major earner which probably indicates that the father would be the major decision maker. But in the majority cases, where financial dependence is on other family members, choice of feeding mode would not be made by the young mother or young parents. Prior studies have also identified cultural beliefs, social and family pressure as the reasons for early cessation of exclusive breastfeeding [7, 8]. Whereas, studies have also shown that the positive example of exclusive breastfeeding practices and its health outcomes among family members and friends, continuity of care and good rapport with the healthcare provider motivates inexperienced as well as experienced mothers to exclusively breastfeed their infants [9–11]. In our study, the successful stories of EBF underpins the importance of positive family environment and support from the healthcare provider. The Governments, international bodies are promoting exclusive breastfeeding as an idealistic goal of pregnancy but as success to EBF is multifactorial, addressing most of them should become a priority. In a rural Indian setting, auxiliary nurses and midwives are the first point of contact for the pregnant ladies and provide antenatal care which ranges from registration of the pregnancy till accompanying the expectant mother to the hospital for delivery to postnatal care. They do counsel the mothers on a range of important issues but counselling of the important family members on the positive health outcome of EBF is of paramount importance and unfortunately, is lacking. Frequency of counselling, method, quality of counselling process definitely needs to be looked at. This mismatch probably explains the poor rate of EBF despite promotions. Repeated episodes of targeted interpersonal communication involving significant family members would probably increase the knowledge, skill to breastfeed the baby and immediate support, all an environment for positive change [12]. Mothers perception of baby remaining hungry despite breastfeeding or an insufficient breast milk, or in many instances perceiving continuous crying of the babies as a symptom of insufficient nutrition/thirst, prompted many mothers or family members to take other steps. Though this issue can be argued on the basis of poor maternal nutritional status, as most of our study population belong to the poor socio-economic stratum, and 22 mothers had a BMI of less than 18.5 Kg/m^2^, evidence suggests that except for severely malnourished status, maternal nutritional status hardly has any effect on breast milk production and composition [13]. Many authors have actually argued this as a cultural phenomenon, where less milk secretion is a result of less nipple stimulation due to start of supplemental feeding [14]. Whereas, many authors believe it as a complex synergistic interaction between socio-economic influences, cultural influences, mothers’ psychological state and feeding management [15, 16].

### Limitations

The study allowed us to follow the mother-child dyads for 42 days postpartum period on a weekly basis, using 24-h maternal recall to capture the feeding practices. However, as daily variation in feeding practices were very much unlikely for such small children and 24 h maternal recall is a practiced method of data collection [17], the reported variations in this study were highly reflective of the current feeding practices.

## Conclusions

Study findings highlight the importance of preparation of family members, as the elders are as important when preparing the mother for the arrival of a newborn. However, the present counselling process under maternal and child health program is more focused towards the pregnant women. Hence, we conclude that an integrated family centered narrative counselling method, targeting the time period for preparing the expectant mother and family will be the keys for a better infant feeding outcome, a multi-layered complex issue [18–21].

### Ethical statement

This study had been approved by Institutional Ethics Committee of ICMR-National Institute of cholera and enteric diseases (ICMR – NICED), Kolkata, India (No- A -I/2017-IEC). All the participants have been consented (written) prior recruiting them in the study.

## Data Availability

The datasets used and/or analysed during the current study are available from the corresponding author on reasonable request.
